# Tryptophan: A Rheostat of Cancer Immune Escape Mediated by Immunosuppressive Enzymes IDO1 and TDO

**DOI:** 10.3389/fimmu.2021.636081

**Published:** 2021-02-23

**Authors:** Minah Kim, Petr Tomek

**Affiliations:** Auckland Cancer Society Research Centre, Faculty of Medical and Health Sciences, University of Auckland, Auckland, New Zealand

**Keywords:** IDO1, TDO, inhibitors, immunotherapy, tryptophan, kynurenine, AhR, System L

## Abstract

Blockade of the immunosuppressive tryptophan catabolism mediated by indoleamine 2,3-dioxygenase 1 (IDO1) and tryptophan 2,3-dioxygenase (TDO) holds enormous promise for sensitising cancer patients to immune checkpoint blockade. Yet, only IDO1 inhibitors had entered clinical trials so far, and those agents have generated disappointing clinical results. Improved understanding of molecular mechanisms involved in the immune-regulatory function of the tryptophan catabolism is likely to optimise therapeutic strategies to block this pathway. The immunosuppressive role of tryptophan metabolite kynurenine is becoming increasingly clear, but it remains a mystery if tryptophan exerts functions beyond serving as a precursor for kynurenine. Here we hypothesise that tryptophan acts as a rheostat of kynurenine-mediated immunosuppression by competing with kynurenine for entry into immune T-cells through the amino acid transporter called System L. This hypothesis stems from the observations that elevated tryptophan levels in TDO-knockout mice relieve immunosuppression instigated by IDO1, and that the vacancy of System L transporter modulates kynurenine entry into CD4+ T-cells. This hypothesis has two potential therapeutic implications. Firstly, potent TDO inhibitors are expected to indirectly inhibit IDO1 hence development of TDO-selective inhibitors appears advantageous compared to IDO1-selective and dual IDO1/TDO inhibitors. Secondly, oral supplementation with System L substrates such as leucine represents a novel potential therapeutic modality to restrain the immunosuppressive kynurenine and restore anti-tumour immunity.

## Introduction

The human immune system can recognise and eradicate tumour cells. Thus, the immunity plays a key role in reducing cancer incidence (1). However, the immunity is a double-edged sword. Elimination of immune-sensitive tumour cells drives evolution of tumours towards the immune-resistant phenotype in a process called cancer immune-editing (2). This, in turn, leads to malignant and clinically apparent cancer. It is of utmost importance to identify and silence tumoural immune escape mechanisms to restore the patient’s anti-tumour immunity. Inhibiting the T-cell regulatory checkpoints, such as the programmed cell death protein 1 (PD-1) axis, by monoclonal antibodies have shown remarkable clinical responses (3). Some patients on the anti-PD-1 inhibitors experience durable tumour regressions but a sizeable fraction of patients do not benefit from these agents (4). This has triggered a search for mechanisms that could be modulated to optimise cancer patients’ responses to immune checkpoint inhibitors (5, 6). One of the key candidates are enzymes IDO1 and TDO that accelerate immunosuppressive tryptophan catabolism along the kynurenine pathway (7).

Initially thought of as a “holy grail” for potentiating cancer immunotherapy, the disappointing outcomes of IDO1 inhibitors in clinical trials (8–10) have generated scepticism (11–13). However, the evidence indicates that the concept of blocking the kynurenine pathway for potentiating immunotherapy is sound and holds true in preclinical models. It is likely that the most optimal therapeutic approaches to silence the kynurenine pathway have not yet been identified. We envision that improved understanding of the immunosuppression induced by the kynurenine pathway will yield insights into optimised therapeutic strategies.

In this perspective, we summarise the current knowledge about the mechanisms mediating immunosuppression by IDO1/TDO, propose a novel immune-regulatory function for the IDO1/TDO’s substrate tryptophan, and discuss the potential impact of this novel function on therapeutic strategies to block the immunosuppressive tryptophan catabolism.

## Immunosuppressive Tryptophan Catabolism

Mammals metabolise more than 90% of tryptophan *via* the kynurenine pathway (KP) (14). The resulting tryptophan metabolites are involved in essential biological processes such as immune regulation, energy metabolism and production of an important enzyme co-factor NAD (15–17). The first and rate limiting step of the KP, tryptophan oxidation, is catalysed by intracellular enzymes IDO1 and TDO (18). There is also a third enzyme called IDO2, but its low catalytic activity suggests that its principal role is unlikely to oxidise tryptophan (19, 20).

Although both IDO1 and TDO catalyse the identical biochemical reaction, their physiological roles differ. IDO1 regulates peripheral immunity (21) and is induced by pro-inflammatory molecules including type I and II interferons, TNF-α, lipopolysaccharide, and prostaglandin E (22–25) in a wide range of cells including myeloid cells, fibroblasts, and cancer cells (26). Due to its inducible nature, IDO1 is absent in most tissues except for the sites where the body is exposed to non-self antigens such as lung, intestine, pregnant placenta, and lymphoid organs (27). In contrast, the evolutionarily older TDO is primarily expressed in the liver where it degrades excess dietary tryptophan (28–30). TDO can be induced by corticosteroids and activated by excess tryptophan (31–33).

The immunosuppressive role of the KP came to the fore in 1998 as a mechanism that confers an allogeneic foetus the ability to evade destruction by the mother’s T-cells (34). Researchers soon realised that the powerful immunosuppressive effect of KP could be co-opted by cancers to escape immune destruction (35, 36). It is now well established that a wide range of different cancer types thrive on accelerated tryptophan catabolism (27, 37–39). However, the mechanisms whereby KP regulates immunity are not completely understood.

### Mechanisms Involved in Immunosuppression Mediated by Accelerated Tryptophan Catabolism

**Figure 1:**
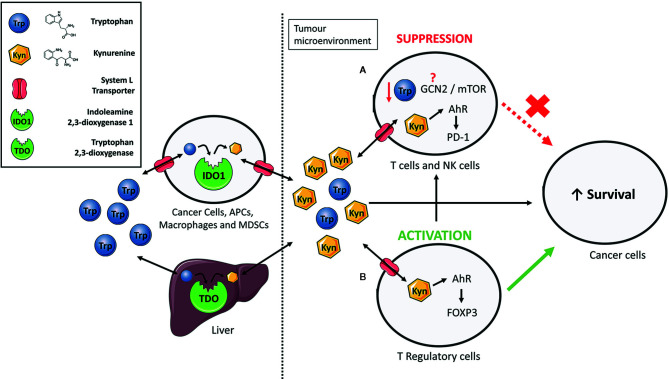
The immunosuppressive functions of IDO1/TDO-mediated tryptophan catabolism. Extrahepatic and hepatic cells express indoleamine 2,3-dioxygenase 1 (IDO1) and tryptophan 2,3-dioxygenase (TDO) to consume tryptophan and give rise to numerous bioactive metabolites such as kynurenine. Elevated expression of IDO1 or TDO, such as in cancer, increases the relative kynurenine levels while reducing tryptophan content. Kynurenine enters cells *via* System L transporters. **(A)** Increased kynurenine levels inhibit proliferation of T-cells and natural killer (NK) cells by interacting with aryl hydrocarbon receptor (AhR) to express programmed cell death protein 1 (PD-1). Previous studies have suggested the involvement of the general control non-deprepressible-2 (GCN2) kinase and mammalian target of rapamycin (mTOR) in proliferation inhibition but the exact mechanism through which this occurs still remains unresolved. **(B)** Kynurenine induces differentiation of naïve CD4^+^ T-cells to immunosuppressive T-regulatory cells by activation of AhR and induction of the FoxP3 transcription factor. Taken together, an immune suppressed tumour microenvironment is created that promotes survival of cancer cells.

As KP is a metabolic pathway, its immune regulatory role has been attributed mainly to tryptophan deprivation and the accumulation of the kynurenine pathway metabolites (**Figure 1**) (40). However, IDO1 also has a non-enzymatic function in which the enzyme acts as a signalling protein in a non-canonical NF-κB pathway driven by immunosuppressive cytokine TGF-*β* (41–43). The contribution of each of these mechanisms to the immune regulation is actively discussed in the research community but accumulating evidence suggests that kynurenine is likely the main culprit (44). Increased kynurenine levels have been associated with reduced function of Natural Killer cells (45) and T-cells (46, 47). Mechanistically, kynurenine’s immune regulatory function is primarily linked to a transcription factor called aryl hydrocarbon receptor (AhR). Binding of kynurenine to AhR induces differentiation and activation of immunosuppressive T-regulatory cells (48–53), contributes to the recruitment of tolerogenic myeloid cells such as macrophages (54), and increases expression of the immune checkpoint molecule PD-1 on tumour-specific CD8^+^ T-cells (**Figure 1**) (55).

In contrast, the role of tryptophan deprivation in immune regulation is somewhat controversial. An earlier study has demonstrated that tryptophan deprivation inhibits proliferation and induces apoptosis in T-cells (56). But these experiments have been mostly carried out in the dish where tryptophan can get depleted. As tumours accelerating tryptophan catabolism still contain sufficient levels of tryptophan (57–59), it is likely that tumoural tryptophan levels cannot reach levels sufficiently low to activate the stress response pathways to low amino acid levels such as the general control non-derepressible-2 (GCN2) and the mammalian target of rapamycin (mTOR) (**Figure 1**). The key protagonist involved in regulation of T-cell responses to tryptophan deprivation was initially suspected to be the GCN2 kinase (60). GCN2 mediates conserved stress response pathway to amino acid deprivation in eukaryotes and was shown to be activated in T-cells in response to tryptophan deprivation (60, 61). However, this finding was recently challenged by two studies. Sonner and colleagues demonstrated no difference in the level of immune responses between the GCN2-proficient and GCN2-deficient T-cells against B16 melanomas (59). Similarly, tryptophan deprived T-cells ceased proliferation even in the absence of the *GCN2* gene (62). The unlikely role of GCN2 as a low tryptophan sensor in immune cells is further corroborated by studies questioning the canonical role of GCN2 as a sensor of amino acid deficiency in mammals. GCN2 stress response pathway has been widely accepted as a mechanism for maintenance of amino acid homeostasis by controlling the feeding behaviour of omnivores (63). However, more recent studies challenged this paradigm as no significant difference in feeding behaviour was observed between the GCN2-deficient and GCN2-proficient mice that were amino acid-deprived (64). Complementary to GCN2, the mTOR senses amino acid sufficiency (65) and was proposed as a mediator of cellular stress response to low tryptophan levels. Metz and colleagues reported the repression of mTOR kinase activity in tryptophan-deprived HeLa cells, which eventually led to cell cycle arrest and apoptosis (66). It is not yet understood if mTOR could sense low tryptophan levels in immune cells.

The collective evidence accumulated to date tends to favour the conclusion that tryptophan serves primarily as a kynurenine precursor rather than inducing stress by its deprivation in vivo (67). However, a recent study by Schramme and colleagues provides a clue to a new immune regulatory function of tryptophan (57). In this study, elevated (5 to 10-fold) systemic tryptophan levels reaching 500 µM in TDO2- knockout mice overturned tumoural immune suppression induced by IDO1. Consequently, anti-PD1 immune checkpoint therapy alone was sufficient to impede the growth of IDO1-proficient MC38 colon tumours in these TDO2-knockout mice (57). That is a striking observation but how can it be explained mechanistically? How can elevation in circulating tryptophan levels overcome IDO1-mediated immunosuppression in the tumour? We posit that elevated tryptophan levels reverse the immunosuppression by outcompeting kynurenine for entry into T-cells through a shared amino acid transporter.

### Tryptophan and Kynurenine: Transporter Competitors

IDO1 and TDO are intracellular enzymes; hence they require the cells to import tryptophan from the extracellular space. Import of large amino acids such as tryptophan typically occurs through the transporter called System L (68, 69). System L transporters are heterodimeric transmembrane proteins comprising a glycoprotein heavy chain (CD98) and a catalytic light chain (LAT1 or LAT2). Kynurenine can also be transported into cells through the System L transporters (70). It has been suggested that the transporters are bidirectional and can exchange tryptophan for kynurenine in cancer cells (71). This would explain the ability of cancer cells to siphon tryptophan from the microenvironment and enrich it with kynurenine to create an immunosuppressive milieu. As System L transports a broad range of amino acids, the transporter substrates compete with each other, and the probability of interacting with the transporter depends on the relative levels of the specific amino acid and their respective affinity for the transporter (72).

**Figure 2:**
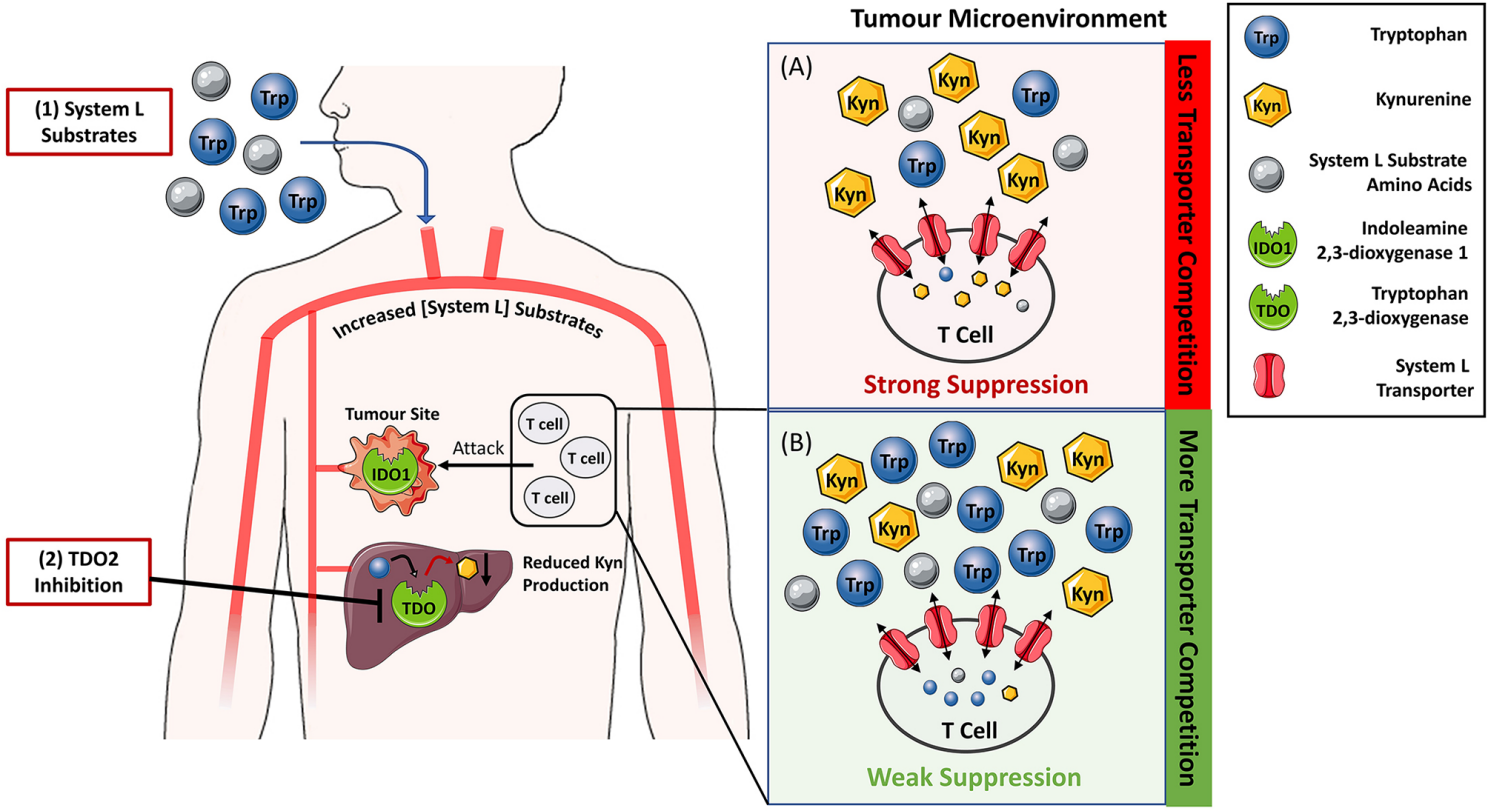
Reversing IDO1/TDO-mediated immunosuppression by increasing the levels of System L transporter substrates to limit kynurenine entry into T-cells. **(A)** In tumour microenvironment rich in indoleamine 2,3-dioxygenase 1 (IDO1) and tryptophan 2,3-dioxygenase 2 (TDO), the tryptophan to kynurenine ratio is typically low leading to the suppression of T-cell activity and tumour killing. Blockade of TDO enzymatic activity by small molecule inhibitors (2) and/or supplementation with System L substrates such as leucine (1) is expected to increase their blood levels. Hence, the ratio of System L substrates to kynurenine in the tumour microenvironment **(B)** will also be increased. Elevated System L substrate levels competitively inhibit kynurenine entry into T cells so that T cell suppression is reduced.

This observation indicates that the relative ratio of tryptophan to kynurenine will influence the amount of kynurenine entering T-cells. Therefore, tryptophan concentration can be viewed as a rheostat that modulates kynurenine entry into T-cells and the resulting immunosuppression (**Figure 2**). This concept is supported by literature evidence. The vacancy of System L transporter influences the ability of kynurenine to enter the CD4+ T-cells and activate AhR (73). Further, both low tryptophan levels and kynurenine accumulation appear to be pivotal for immunosuppression mediated by tryptophan catabolism (47), and tryptophan supplementation reverses the proliferation arrest of IDO1-mediated tryptophan deprivation in T-cells (60, 74). Tryptophan acting as a rheostat of kynurenine-mediated immunosuppression has two important therapeutic implications. Firstly, it can aid to resolve the conundrum whether the IDO1-selective, TDO-selective or dual IDO1/TDO inhibitors would be the most optimal therapeutic agents to block KP. Secondly, it can serve as a basis for a new approach to silence kynurenine-mediated immunosuppression by oral supplementation with System L transporter substrates. We will discuss these two areas in the following section.

## Discussion

### Efficient Silencing of Kynurenine Pathway May Require Only TDO-Selective Inhibitors

Many cancer types co-opt IDO1, TDO or both enzymes (27, 39, 75) to accelerate tryptophan catabolism and escape immune destruction. Upregulation of IDO1 or elevated kynurenine levels associate with poor patient outcomes (37, 76–79) and resistance to immune checkpoint therapy such as anti-PD-1 inhibitors (80, 81). Moreover, anti-PD-L1 therapy promotes tryptophan catabolism as a consequence of IDO1 upregulation by IFN-*γ* secreted by re-invigorated tumour-infiltrating lymphocytes (82). These observations provide a strong mechanistic rationale for combining the tryptophan catabolism blockade with immune checkpoint inhibitors and potentially other cancer immunotherapies. Targeting the KP also offers certain advantages over targeting cell surface immune-regulatory molecules such as PD-1. Proteins on the KP including IDO1 and TDO are intracellular enzymes (40, 83) that harbour active sites easily targetable with inexpensive and non-immunogenic small molecules. Such molecules are stable on storage, can be administered orally, and penetrate into the brain. This is in contrast to immune checkpoint molecules such as PD-1 that do not catalyse a biochemical reaction hence lack an active site, and typically require costly antibodies for therapeutic modulation. Due to their size and instability, antibodies need to be administered by injection and cannot pass the blood brain barrier. Of note, small molecules that disrupt PD-1/PD-L1 interaction are currently in development (84, 85).

Major effort has been devoted to disable tryptophan catabolism using IDO1 inhibitors (7, 86, 87). Whilst IDO1 inhibitors can boost immunotherapy in mouse cancer models (88–91), the most advanced IDO1 inhibitor Epacadostat (INCB024360) could not potentiate anti-PD1 inhibitor pembrolizumab in a recent Phase III trial involving about 700 advanced melanoma patients (9, 92). Reasons for this negative outcome are unclear and discussed extensively elsewhere (11–13, 93, 94) but poor target engagement, compensatory expression of TDO or IDO2, dose-limiting toxicities or the lack of selection for IDO1-positive patients are likely one of the contributing factors.

Unlike IDO1 inhibitors, no TDO-specific inhibitor has yet reached clinical trials. This is not surprising given the paucity of potent TDO-selective inhibitors. High affinity TDO inhibitors seems to be much more difficult to develop than IDO1 inhibitors. That is likely because the TDO’s active site is less flexible than that of IDO1 and thus cannot accommodate bulky ligands (7, 95). As a consequence, the majority of TDO-specific inhibitors [reviewed in (7, 96)] mimic tryptophan (28, 36, 97–100). Currently, the most promising TDO inhibitors appear to be the derivatives of IDO1-specific clinical candidate Navoximod (100) reported by Genentech, and PF06845102 (57) developed by iTeos Therapeutics. These inhibitors display submicromolar potencies, up to 100-fold TDO selectivity over IDO1, good metabolic stability and ability to raise systemic tryptophan levels in mice (57, 100). PF06845102 has also been shown to potentiate anti-tumour activity of the anti-CTLA4 immune checkpoint inhibitor in a mouse model of colorectal cancer (57). These promising data support further development of next-generation TDO inhibitors.

The hypothesis presented in this perspective suggests that TDO inhibition can be advantageous to IDO1 inhibition. Firstly, TDO inhibition is expected to indirectly inhibit the immunosuppressive action of IDO1 by raising the systemic levels of tryptophan and limiting entry of IDO1-generated kynurenine into T-cells (**Figure 2**). This concept assumes relatively low contribution of non-enzymatic signalling function of IDO1 to the overall immunosuppression. Secondly, unlike IDO1 inhibitors, TDO inhibitors do not require tumoural TDO expression because TDO is constitutively expressed in the liver. This is clearly demonstrated in studies where TDO2-knockout mice but not IDO1-knockout mice have markedly increased plasma tryptophan levels compared to their respective wild-type counterparts (29, 57). Therefore, TDO inhibition is anticipated to silence kynurenine-mediated immunosuppression in a greater subset of patients. The potential toxicity of hepatic TDO blockade in humans still remains unresolved. However, the absence of serious clinical pathologies of a woman diagnosed with hypertryptophanaemia due to TDO deficiency (101) suggests that TDO inhibition will be well tolerated in humans.

As cancers can express both IDO1 and TDO, the industry has been pursuing the development of dual IDO1/TDO inhibitors. This concept has not yet been supported by strong evidence but some dual inhibitors are in preclinical development or Phase I trials (40, 102, 103) including Navoximod (89) which was originally thought to be an IDO1-selective inhibitor. Similarly to IDO1 inhibitors, we contend that development of dual inhibitors may be unnecessary. However, it cannot be excluded that IDO1 inhibition will be needed to complement TDO blockade. Inhibition of hepatic TDO may not increase tryptophan levels significantly in humans. Further, it is possible that kynurenine enters cells through a transporter other than System L or triggers the immunosuppressive effect in the absence of secretion from the IDO1/TDO-expressing cells.

### Can High-Dose Amino Acid Supplementation Reverse Kynurenine Mediated Immunosuppression and Potentiate Immunotherapy?

We propose that any strategy that safely increases the levels of circulating System L substrates to out-compete kynurenine has potential to reverse the IDO1/TDO mediated immunosuppression. One additional possibility to TDO inhibition is oral supplementation with amino acids that are System L substrates (**Figure 2**) such as leucine, isoleucine, valine, phenylalanine, tyrosine, tryptophan, methionine, or histidine. Whilst tryptophan supplementation emerges as a possibility, it is unlikely to increase systemic tryptophan levels because hepatic TDO efficiently breaks down excess tryptophan. This is consistent with the study of Schramme et al. showing that three-fold increase of tryptophan in the diet from 0.06 to 0.18% did not increase circulating tryptophan levels of the mice (57). Further, tryptophan supplementation (30 mg per mouse) did not significantly impede growth of mouse CT26 colon tumours (104). On the other hand, there are preclinical data showing that leucine, a high affinity substrate of System L, limits System L-mediated entry of kynurenine into brain (105). There are no data available to show if high-dose dietary supplementation with leucine or any other amino acid would translate into improved tumour control or blockade of kynurenine-mediated immunosuppression. However, the above-mentioned study strongly supports the feasibility of limiting kynurenine transport *in vivo* at leucine doses that are well tolerated by an organism.

The safety of high dose amino acid supplementation raises a potential concern. It is generally assumed that amino acids do not pose serious health hazards as they are natural substances produced endogenously and part of human diet and supplements (106). Perhaps not surprisingly, toxicities associated with high dose amino acid supplementation to mammals differ significantly (107) but leucine appears to be the least toxic amino acid. Oral or intravenous supplementation of leucine (5 g–6 g) increased systemic leucine levels in humans in the absence of overt toxicities (108, 109). Similarly, as stated in the preceding paragraph, elevated leucine levels sufficient to prevent kynurenine transport are well tolerated by mice. This is in contrast to methionine and histidine which, at high doses, appear to be one of the most toxic amino acids to humans (110–112). However, the toxicity of a substance depends on its dose. It is therefore likely that even the seemingly most toxic amino acids may prevent kynurenine entry into T-cells at levels which are well tolerated by an organism. Experimental studies will be necessary to rigorously investigate this concept and determine which amino acids and at what doses will provide therapeutic benefit, if any.

Overall, the therapeutic supplementation with a high dose of amino acids with the intent to inhibit kynurenine-mediated immunosuppression appears like a highly feasible and exciting research prospect. It offers a simple and economical alternative to synthetic drugs inhibiting tryptophan catabolising enzymes or downstream kynurenine targets such as AhR.

## Summary

The disappointing outcome of the Phase III trial of Epacadostat (9) has stimulated search for alternative approaches to silence the KP. Such approaches include kynurenine depletion by kynureninase (44), small molecule inhibitors to the kynurenine’s downstream target AhR (81, 94), and perhaps we will see small molecule inhibitors to a recently discovered tryptophan metabolising enzyme IL4I1 that produces AhR agonists (113). This perspective proposes a novel function for the IDO1’s substrate tryptophan that could lead to an additional therapeutic strategy to block KP. We posit that tryptophan acts as a rheostat of kynurenine-mediated immunosuppression, *i.e.*, high tryptophan to kynurenine ratio limits kynurenine’s entry into immune T-cells through the shared System L amino acid transporter.

Therefore, increasing circulating levels of System L substrates can relieve kynurenine-induced immunosuppression. One way to achieve this is *via* inhibition of hepatic kynurenine pathway by TDO inhibitors. This supports the development of TDO-selective inhibitors that, unlike IDO1 inhibitors, are not contingent on tumoural TDO expression. Alternatively, kynurenine can be out-competed by therapeutic supplementation of amino acids such as leucine which is a high-affinity System L substrate. Leucine supplementation appears highly feasible. Leucine has low toxicity to mammals and was shown to block kynurenine entry into the mouse brain. If confirmed, we envision the amino acid supplementation strategy will enrich the armamentarium of therapeutic approaches modulating KP, and increase the likelihood of realising the prospect of silencing the KP for cancer immunotherapy.

## Data Availability Statement

The original contributions presented in the study are included in the article/supplementary material. Further inquiries can be directed to the corresponding author.

## Author Contributions

PT conceived, designed and supervised the study. MK drafted the manuscript and figures. All authors contributed to the article and approved the submitted version.

## Funding

PT and MK acknowledge support from Health Research Council New Zealand through the Emerging Researcher First Grants 17/586 awarded to PT. PT acknowledges additional support from Auckland Medical Research Foundation Project grant 1120009 awarded to PT, School of Medical Sciences at the University of Auckland in New Zealand and Auckland Cancer Society Research Centre. The funding bodies had no role in design of this study, decision to publish and preparation of the manuscript.

## Conflict of Interest

The authors declare that the research was conducted in the absence of any commercial or financial relationships that could be construed as a potential conflict of interest.

## Abbreviations

AhR, Aryl hydrocarbon Receptor; GCN2, General control non-derepressible 2; IDO1, Indoleamine 2,3-dioxygenase 1; KP, Kynurenine Pathway; mTOR, Mammalian target of rapamycin; PD-1, Programmed cell death protein 1; TDO, Tryptophan 2,3-dioxygenase.
